# Severe community onset healthcare-associated *Clostridium difficile* infection complicated by carbapenemase producing *Klebsiella pneumoniae* bloodstream infection

**DOI:** 10.1186/1471-2334-14-475

**Published:** 2014-09-01

**Authors:** Simone Giuliano, Maurizio Guastalegname, Miryam Jenco, Andrea Morelli, Marco Falcone, Mario Venditti

**Affiliations:** Department of Public Health and Infectious Diseases Policlinico Umberto I, “Sapienza” University of Rome, Viale del Policlinico 155, 00161 Rome, Italy; Department of Anesthesiology and Intensive Care, “Villa San Pietro-Fatebenefratelli” Hospital, Rome, Italy; Department of Anesthesiology and Intensive Care, “Sapienza” University of Rome, Rome, Italy

**Keywords:** *Clostridium difficile*, *Klebsiella pneumoniae*, KPC, Bloodstream infections, Intestinal flora, Fidaxomicin

## Abstract

**Background:**

*Clostridium difficile* infection (CDI) and *Klebsiella pneumoniae* carbapenemase producing-*Klebsiella pneumoniae* (KPC-Kp) bloodstream infection (BSI) are emerging health-care associated (HCA) diseases of public health concern, in terms of morbidity, mortality, and insufficient response to antibiotic therapy. Both agents can be acquired in the hospital but clinical disease can develop in a community setting, after discharge. We report here a putative link between the above-mentioned healthcare associated infections that appeared as a dramatic community onset disease with a fulminant fatal outcome.

**Case presentation:**

We describe the case of a 63 year old man affected by severe CDI. Even though the patient underwent 72 hours of standard CDI antibiotic treatment, the clinical course was complicated by toxic megacolon and KPC-Kp BSI. The patient died 24 hours after total colectomy.

**Conclusion:**

The impact of HCA-BSIs in complicating CDI is still unknown. Intestinal inflammatory injury and disruption of intestinal flora by standard antibiotic treatment could be responsible for promoting difficult-to-treat infections in CDI. By preserving intestinal flora, Fidaxomicin could have a crucial role in preventing BSIs complicating severe CDI.

## Background

The impact of health-care associated (HCA) bloodstream infection (BSI) in complicating *Clostridium difficile* infection (CDI) is still unknown. We describe for the first time a case of community onset healthcare-associated severe CDI complicated by *Klebsiella pneumoniae* Carbapenemase producing *Klebsiella pneumoniae* (KPC-Kp) BSI. CDI and KPC-Kp BSI are recently emerging as two of the more concerning HCA diseases in terms of morbidity, mortality, and inefficient response to antibiotic therapy. A relation between the above-mentioned infections have never been reported until now.

## Case presentation

We report the case of a 63 year old man, who was admitted on the 6th of February 2014 to the Intensive Care Unit (ICU) of a 1300-bed teaching Institution in Rome because of septic shock syndrome. His past medical history was remarkable for high blood pressure, multi-infarct dementia, Parkinson’s disease and depressive disorder. The patient had been hospitalized on the 15th of January 2014 in a peripheral center for a febrile syndrome due to a urinary tract infection caused by *Escherichia coli*. During his hospital stay, he underwent a ten-day course of antimicrobial therapy with intravenous ciprofloxacin and was discharged on the 28th of January on treatment with oral Cefditoren pivoxil. On the 3rd of February, the patient was readmitted to the same hospital because of confusion, fever and diarrhea. *Clostridium difficile* infection (CDI) test was performed (Alere™ C. DIFF QUICK CHECK COMPLETE for simultaneous detection of both glutamate dehydrogenase antigen and toxin A and B). Oral vancomycin 500 mg q6h, intravenous metronidazole 500 mg q6h and intravenous Piperacillin/tazobactam 4.5 g q8h were immediately started. After 72 hours of persistent deterioration despite standard antibiotic therapy administration, the patient was transferred to the ICU of our hospital. At the admission the patient was unconscious (Glasgow Coma Scale of 3), presenting with ileus and septic shock syndrome. Oro-tracheal intubation and fluid resuscitation were performed followed by intravenous norepinephrine. Tigecycline was empirically added to the combination antimicrobial therapy. Three blood cultures were drawn and blood test showed 24000 white blood cells/μl, creatinine 3.6 mg/dl, albumin 2.2 g/dl, lactate 4 mmol/l. Computed tomography scans demonstrated findings compatible with toxic megacolon. A total colectomy was performed but the patient died 24 h after the surgical intervention. All blood cultures drawn at the time of admission to the ICU were positive for a carbapenem-resistant strain of *Klebsiella pneumoniae* (Vitek 2 system, AST-N089 card bioMérieux, Marcy l’Etoile, France, MICs: Amikacin ≥ 64 mg/l, Amoxicillin/Clavulanic acid ≥ 32 mg/l, Cefepime ≥ 64 mg/l, Cefotaxime ≥ 64 mg/l, Ceftazidime ≥ 64 mg/l, Ciprofloxacin ≥ 4 mg/l, Colistin ≥ 16 mg/l, Ertapenem ≥ 8 mg/l, Fosfomycin ≥ 256 mg/l, Gentamicin 4 mg/l, Imipenem ≥ 16 mg/l, Meropenem ≥ 16 mg/l, Piperacillin/Tazobactam ≥ 128 mg/l, Tigecycline 4 mg/l, Trimethoprim/Sulfamethoxazole ≥ 320 mg/l). Production of KPC enzyme was documented by using a phenotypic test based on the inhibitory activity of boronic acid compounds as reported by Pournaras et al. [1], and carbapenemase producing-*Klebsiella pneumoniae* (KPC-Kp) was identified. In addition, one blood culture was positive for vancomycin-susceptible *Enterococcus faecium*. Histologic examination of the colon confirmed diagnosis of pseudomembraneous colitis.

## Discussion and conclusion

We believe that this case report is of great interest because our patient contemporarily developed two community-onset healthcare associated infections with a rapidly fatal outcome. Carbapenem-resistant Enterobacteriaceae (CRE) are an emerging issue of great public health concern. In our country, the problem is almost completely represented by carbapenem-resistant *K. pneumoniae*. Even though data related to incidence are missing, percentage of invasive *K. pneumoniae* isolates with resistance to carbapenems reportedly ranges between 25% and 50% in Italy [2]. Recently, cases of healthcare-associated carbapenem resistant *K. pneumoniae* BSIs have been reported in Northern Italy [3]. The phenomenon is also well known in Rome [4] and parallels to an epidemiological shift of CDI occurring in our region and consisting of increased disease incidence and mortality rates [5, 6], that are probably due to the spread of the epidemic strain ribotype 027 CD [6–8]. In the present case, because the initial diagnosis was made in a peripheral center, ribotyping test was not performed. We describe, for the first time, a fatal case of severe CDI complicated by KPC-Kp BSI. This led us to question about the role of severe CDI on predisposing to HCA-BSIs, and vice-versa, on the role of HCA-BSIs in determining the prognosis of severe CD colitis. To our knowledge this issue has never been thoroughly evaluated in the literature until now.

We already disserted on the putative correlation between CDI and *Candida* spp. BSI [9]. Analogous mechanisms could be advocated to explain the link between CDI and KPC-Kp BSI. Recently, Perez et al. demonstrated in a mouse model the impact of the administration of antibiotics effective against intestinal flora on promoting the persistence of KPC-Kp colonization of the gastrointestinal tract [10]. Edlund et al., evaluating the ecological disturbances of oral vancomycin following cephalosporin administration in 20 healthy volunteers, showed intestinal *Klebsiella* spp. overgrowth within 3 days of vancomycin treatment [11]. In the present case, KPC-Kp colonization was acquired in a non-ICU setting, an emerging phenomenon already described in our country [12]. Oral vancomycin administration, highly active against the intestinal flora [13], could have been responsible for persistence and overgrowth of KPC-Kp in the gastrointestinal tract. Gut inflammatory injury caused by severe CDI could be considered the “second hit” allowing bacterial translocation and BSI. The role of severe intestinal inflammation predisposing to bacterial mucosal penetration and blood invasion has been already showed in murine and human models [14, 15]. Moreover, *K. pneumoniae* has been demonstrated to be one of the most efficient microorganisms in translocating to extraintestinal sites, presenting a very low bacteremia clearance in the liver and spleen [16].

Colonic inflammation induced by severe CDI and standard CD antibiotic treatment could promote difficult-to-treat infections/colonizations (i.e. KPC-Kp BSI, *Candida* spp. BSI [6], or vancomycin resistant-enterococci intestinal colonization [17]. Fidaxomicin, a newly licensed macrocyclic antibiotic recently approved to treat CDI, is characterized by a narrow spectrum of activity almost limited to CD and consequent preservation of intestinal flora compared to vancomycin [13]. Preservation of intestinal flora could represent a useful therapeutic option not only in reducing CDI recurrence rate [9], but also in preventing BSIs secondary to severe CDI.

In conclusion, clinical impact of BSIs on complicating CDI has not been systematically evaluated until now. Fidaxomicin could represent a useful therapeutic option in preventing difficult-to-treat BSIs secondary to severe CDI. Studies are necessary to validate this hypothesis.

## Consent

Written informed consent was obtained from the next of a kin of the patient for publication of this Case report. A copy of the written consent is available for review by the Editor of this journal.
